# Clinically significant hypoglycaemia in hospitalised adults: A multicentre study of 1205 episodes in the United Kingdom

**DOI:** 10.1111/dme.70331

**Published:** 2026-04-20

**Authors:** Charles Page, Mariam Idrissi, Sam Sherratt‐Mayhew, Aashritha Buchipudi, Amanda Ling Jie Yee, Kalyaani Persad, Aqeelah Khatoon, Amrit Rakkar, Amynta Arshad, Alexandra Lubina Solomon, Rajeev Raghavan, Lakshmi Rengarajan, Ahmed Iqbal, Pratik Choudhary, Aspasia Manta, Punith Kempegowda, DEVI Collaboration

**Affiliations:** ^1^ Birmingham Medical School, College of Medicine and Health University of Birmingham Birmingham UK; ^2^ Warwick Hospital Warwick UK; ^3^ Department of Applied Health Sciences, School of Health Sciences, College of Medicine and Health University of Birmingham Birmingham UK; ^4^ Russells Hall Hospital Dudley UK; ^5^ The Royal Wolverhampton NHS Trust Wolverhampton UK; ^6^ Walsall Manor Hospital Walsall UK; ^7^ Division of Clinical Medicine, School of Medicine and Population Health University of Sheffield Sheffield UK; ^8^ Department of Population Health Sciences, George Davies Centre University of Leicester Leicester UK; ^9^ Department of Diabetes and Endocrinology Queen Elizabeth Hospital Birmingham, University Hospitals Birmingham NHS Foundation Trust Birmingham UK

**Keywords:** diabetes complications, hypoglycaemia, inpatient care, mortality, quality improvement, risk factors

## Abstract

**Introduction and Objectives:**

Inpatient hypoglycaemia is a serious complication of diabetes therapy, yet real‐world data on clinically significant episodes remain limited. The objectives of this study are (1) to describe the clinical and biochemical characteristics of clinically significant inpatient hypoglycaemia across multiple NHS sites, (2) to assess adherence to Joint British Diabetes Societies hypoglycaemia management guidelines, (3) to evaluate the applicability of International Hypoglycaemia Study Group (IHSG) classifications in hospitalised people with diabetes and (4) to identify gaps in care processes amenable to quality improvement (QI) interventions.

**Methods:**

We conducted a retrospective cohort study across 11 UK centres, including all clinically significant hypoglycaemic episodes (IHSG Levels 2 and 3) between October 2023 and December 2024. Data on demographics, precipitants, clinical features, treatment, outcomes and subsequent therapeutic adjustments were analysed.

**Results:**

We identified 1205 episodes from 674 people (mean age 72 years; 54.5% male; 74.6% with type 2 diabetes; median Charlson Comorbidity Index 6). Common precipitants included intercurrent illness (44.7%), fasting/missed meals (40.7%) and medication dosing errors (16.7%). Inpatient mortality occurred in 2.9% of Level 2 and 15.2% of Level 3 episodes. Episode characteristics varied by sex, diabetes type and centre. The IHSG classification system effectively stratified the risk of adverse outcomes.

**Conclusions:**

Clinically significant inpatient hypoglycaemia is associated with high mortality and substantial variation in care. The IHSG framework reliably predicts poor outcomes and may serve as a valuable framework for standardised risk stratification and service evaluation. Identification of common, modifiable risk factors highlights opportunities for systematic QI and surveillance.

**What's new?**

- What is known about this subject?
  - Hypoglycaemia is associated with high mortality rates, underscoring the urgent need for improved surveillance and prevention strategies.
  - Guidelines from various organisations, including the Joint British Diabetes Societies, provide management recommendations, but evidence on guideline implementation and outcomes of clinically significant hypoglycaemia is limited.
  - Existing data sources and studies lack granularity, often reporting only event frequencies or modelling associations with outcomes without analysing individual hypoglycaemic episodes in detail.
- What is the key question?
  - How is clinically significant inpatient hypoglycaemia currently characterised and managed across the United Kingdom, and what gaps in classification, guideline adherence and care processes exist that could inform quality improvement?
- What are the new findings?
  - We identified common, modifiable risk factors, including intercurrent illness, fasting and medication dosing errors, providing clear targets for intervention and prevention.
  - Outcomes varied significantly between centres and demographic groups, highlighting healthcare inequities and the need for quality improvement initiatives.
  - By validating the IHSG classification system in a large, real‐world inpatient cohort, our study strengthens its role as a global framework for hypoglycaemia reporting and benchmarking.
- How might this impact clinical practice in the foreseeable future?
  - It will encourage improved adherence to care standards, systematic auditing and targeted risk‐reduction strategies.

## 1 INTRODUCTION

Hypoglycaemia remains a major contributor to diabetes‐related morbidity and mortality, particularly in the hospital setting, where people with diabetes are exposed to dynamic clinical, pharmacological and nutritional conditions.^1^ The clinical spectrum of hypoglycaemia ranges from asymptomatic biochemical episodes to life‐threatening events, including coma and death.^2^ Despite this, hypoglycaemia remains undercharacterised in the inpatient population.

Current national guidance in the United Kingdom, such as the Joint British Diabetes Society (JBDS) guidelines, provides detailed recommendations for the management of inpatient hypoglycaemia.^3^ However, there is a paucity of real‐world evidence describing clinically significant hypoglycaemic episodes, particularly concerning precipitating factors, treatment adherence and patient outcomes.^4^

The National Diabetes Inpatient Safety Audit, a UK‐wide data collection initiative, reports broad frequencies of diabetes‐related emergencies but lacks the granularity required to evaluate the context, management and consequences of discrete hypoglycaemic events.^5^ Other studies have examined hospitalisations during which hypoglycaemia occurred but often fail to isolate individual episodes for detailed analysis.^6^ Similarly, predictive modelling studies have linked inpatient hypoglycaemia to adverse outcomes without offering direct insight into causative factors or care quality.^6^, ^7^ At the local level, audit mechanisms suffer from inconsistent methodology, small sample sizes and limited generalisability.^8^

Given that approximately 18% of hospitalised individuals with diabetes experience at least one hypoglycaemic event during admission,^5^ there is a need for a standardised, multicentre surveillance system to capture clinically relevant data. Such a system would enable the identification of modifiable risk factors, support adherence to management guidelines and improve outcomes through shared learning.

The International Hypoglycaemia Study Group (IHSG) has proposed a standardised classification system to facilitate consistent reporting of hypoglycaemia: Level 1 (glucose 3.0–3.9 mmol/L), Level 2 (glucose <3.0 mmol/L, considered clinically significant) and Level 3 (hypoglycaemia associated with severe cognitive impairment requiring external assistance).^9^, ^10^ All glucose levels obtained by capillary self‐monitoring, continuous glucose monitoring (CGM) or plasma glucose measured in the laboratory were used to determine this standardised classification system.^9^ While this classification has been validated in clinical trial settings,^10^ it remains untested in inpatient populations, where factors such as acute illness, polypharmacy and variable monitoring practices may influence both detection and outcomes.

To address these limitations, we developed a multicentre surveillance platform, built on the established Digital Evaluation of Ketosis and Other Diabetic Emergencies (DEKODE) framework for diabetic ketoacidosis.^11^, ^12^ DEKODE prioritises systemic improvements over the short‐term gains typically associated with audit processes.^13^, ^14^ Supported by a centralised database, DEKODE has the potential to function as a national platform, facilitating shared learning across multiple NHS trusts. DEKODE is endorsed by the Society for Endocrinology (SfE), the Association of British Clinical Diabetologists (ABCD) and the NHS Getting It Right First Time programme.

The objectives of this study are (1) to describe the clinical and biochemical characteristics of clinically significant inpatient hypoglycaemia across multiple NHS sites, (2) to assess adherence to JBDS hypoglycaemia management guidelines, (3) to evaluate the applicability of IHSG classifications in hospitalised people with diabetes and (4) to identify gaps in care processes amenable to quality improvement (QI) interventions.

## 2 RESEARCH DESIGN AND METHODS

This retrospective, observational, multicentre study was conducted at the University of Birmingham, UK, as part of a quality improvement initiative. Data were collected from 11 acute care hospitals across the United Kingdom (hereafter referred to as Sites A–K), representing a mix of secondary and tertiary care institutions, varying in size, with a combined catchment population exceeding 3 million individuals. All included sites had established electronic health records systems for hypoglycaemia and joined in the DEKODE hypoglycaemia surveillance model as a service improvement activity following clinical governance approval. Data collection adhered to Caldicott principles and institutional data governance protocols to ensure patient confidentiality and compliance with national data protection regulations.

Hypoglycaemia was defined as per the IHSG criteria as either (a) a nadir glucose level through any mode of measurement including capillary testing, laboratory‐based assessment, glucometers, arterial blood gas samples or patients' CGM data <3.0 mmol/L (Level 2) or (b) any episode requiring external assistance due to altered consciousness or cognitive impairment (Level 3).^6^, ^7^ Inclusion criteria were (1) adults aged ≥16 years; (2) a confirmed diagnosis of diabetes mellitus (type 1, type 2 or other specified types); (3) an inpatient admitted on a hospital ward for at least 6 hours (excluding attendance in Accident and Emergency department); and (4) at least one clinically significant hypoglycaemic episode (IHSG Level 2 or 3) occurring between 1 October 2023 and 31 December 2024. To confirm an episode as IHSG Level 3 hypoglycaemia, data collectors reviewed all clinical documentation and noted the episode—in particular, if further assistance was required, as per the IHSG definition.

People were excluded if the diagnosis of diabetes was uncertain or not documented in the medical record. Where diabetes was confirmed but the subtype (e.g. type 1 or type 2) was not recorded, the episode was included, with diabetes type coded as ‘unspecified’. Data where *n* < 5 were suppressed, as is conventional, to prevent inadvertent identification of individuals.

To avoid duplication and overestimation of events, multiple low glucose readings of <3.0 mmol/L within a 6 hour period were considered part of a single hypoglycaemic episode unless clinical documentation suggested otherwise (e.g. resolution followed by recurrence, which would be formally documented in the Health Record). Data collectors reviewed such instances manually to avoid entering duplicate data. The primary unit of analysis was the hypoglycaemic episode, not the individual person, given the potential for multiple episodes per admission and to capture the index hypoglycaemic episode. All IHSG Levels 2 and 3 episodes were collected across the 11 centres.

### 2.1 Data collection

To facilitate data collection, a standardised, pseudonymised data collection tool was developed in consultation with key stakeholders and end‐users, including frontline clinicians, data governance officers and patient safety representatives. The tool was designed to capture detailed information on demographics, comorbidities, diabetes type and treatment, clinical context, biochemical parameters and outcomes related to each hypoglycaemic episode. Data were manually extracted from electronic and/or paper medical records by data collectors, including resident physicians and senior medical students, who were trained in data collection methods by a senior endocrine consultant. To ensure data quality and fidelity across sites, each hospital designated a site lead who provided oversight, addressed local data collection issues and ensured adherence to protocol standards. All data points were collected and stored in a password‐protected cloud, then transferred to a university‐approved server with appropriate data protection measures in place.

Demographic and clinical data were obtained from patients' medical records. Variables collected included age, sex, self‐identified ethnicity and diabetes type. Ethnicity was categorised into six groups: White, Asian, Black, Mixed, Other and Unknown, consistent with national reporting.^5^ Comorbidity burden was assessed using the Charlson Comorbidity Index (CCI), a validated scoring system for predicting 10‐year mortality risk based on the presence of multiple chronic conditions.^15^ CCI was chosen over other tools due to its ease of calculation during data collection and its widespread standardisation in clinical and research settings.

Potential precipitants of hypoglycaemia were recorded and categorised according to clinical documentation to aid statistical evaluation of differences among imputable factors. This included use of medications known to predispose to hypoglycaemia—specifically antidiabetic agents (e.g. insulin, sulfonylureas) and antipsychotics^16^, ^17^—as well as the presence of contributing clinical factors. Precipitants were grouped into intercurrent illness (e.g. active infection, acute kidney injury, or other acute medical/surgical pathology), fasting or missed meal, including both patient‐initiated fasting (e.g. religious) and iatrogenic causes (e.g. preoperative fasting), incorrect medication dosing, including both self‐administered and clinician‐administered errors, physical exertion and unclear cause (when documentation did not allow attribution). Episodes were additionally flagged where multiple concurrent factors were believed to contribute.

Treatment administered during each hypoglycaemic episode was documented according to categories based on the JBDS guidelines for hypoglycaemia management: not fully documented, oral glucose (including food or sweetened fluids), intravenous dextrose (including any IV glucose solution) or intramuscular glucagon.^3^

Short‐term outcomes were recorded as ongoing inpatient care, escalation to intensive therapy unit (ITU), discharge from hospital and inpatient death. Long‐term outcomes at the end of the hospital admission were classified as simple discharge (routine discharge with no major change in care needs), complex discharge (involving new or increased support from health or social care services), death during admission or other discharge outcome (e.g. transfer to hospice, self‐discharge against medical advice).^18^ Length of stay from the time of the hypoglycaemic episode to discharge or death was also recorded.

Therapeutic adjustments made during or following the hypoglycaemic event were captured and included: medication changes (e.g. discontinuation or dose reduction of sulfonylureas or insulin or changes to other glucose‐lowering agents), specialist diabetes team referral, palliative care initiation or continuation, initiation of CGM and provision of an outpatient glucagon prescription. Medication adjustments were further categorised into the following subgroups: stopping sulfonylureas, reducing the sulfonylurea dose, stopping insulin, reducing the insulin dose and other changes to antidiabetic medications.

### 2.2 Data analysis

Normality was assessed using the Shapiro–Wilk test. Non‐parametric continuous variables were described using medians and interquartile ranges (IQR), with significance assessed via Mann–Whitney *U* tests. Parametric continuous data were reported as means and standard deviations (SD), and significance was assessed using unpaired *t*‐tests. Categorical variables were described as absolute counts (*n*) and proportions (*x*%), with significance assessed using Fisher's exact test. The significance threshold was set at *p* < 0.05. A statistician reviewed the data regularly, and where anomalies were identified, the data were revalidated against original records from local centres by a second team member. All statistical analyses were conducted using Prism GraphPad v10.3.0 and R v4.4.1 (R Studio).

## 3 RESULTS

A total of 1793 Level 2 and 3 hypoglycaemic episodes were collected across 11 NHS centres. Of these, 334 episodes were excluded as they occurred in new admissions (i.e. people presenting from outside the hospital or as outpatients), and a further 254 were excluded as they occurred in individuals either without diabetes or with an uncertain diagnosis. This resulted in a final cohort of 1205 inpatient episodes across 674 people with diabetes (Table 1; Table S1).

**TABLE 1:** Demographics of all included episodes—all results by hospital. All results given as *n* (%), where *n* represents the number of people with diabetes.

	Centre	Overall	A	B	C	D	E	F	G	H	I	J	K
Cohort, *n* (%)		674 (100.0)	197 (29.2)	53 (7.9)	116 (17.2)	113 (16.8)	10 (1.5)	87 (12.9)	^a^	18 (2.7)	^a^	30 (4.5)	45 (6.7)
Sex, *n* (%)	Female	307 (45.5)	81 (41.1)	30 (56.6)	51 (44.0)	59 (52.2)	^a^	40 (46.0)	^a^	6 (33.3)	^a^	10 (33.3)	24 (53.3)
	Male	367 (54.5)	116 (58.9)	23 (43.4)	65 (56.0)	54 (47.8)	7 (70.0)	47 (54.0)	^a^	12 (66.7)	^a^	20 (66.7)	21 (46.7)
Ethnicity, n(%)^b^	White	438 (71.5)	137 (70.3)	12 (26.1)	90 (85.7)	64 (63.4)	8 (80.0)	65 (86.7)	^a^	11 (78.6)	^a^	19 (73.1)	30 (93.8)
	Asian	118 (19.2)	43 (22.1)	26 (56.5)	6 (5.7)	25 (24.8)	^a^	6 (8.0)	^a^	^a^	^a^	5 (19.2)	^a^
	Black	46 (7.5)	9 (4.6)	8 (17.4)	8 (7.6)	12 (11.9)	^a^	^a^	^a^	^a^	^a^	^a^	^a^
	Other/mixed	11 (1.8)	^a^	6 (13.0)	0 (0.0)	^a^	^a^	^a^	^a^	0 (0.0)	^a^	0 (0.0)	0 (0.0)
Diabetes, *n* (%)	Type 1	160 (23.7)	45 (22.8)	9 (17.0)	38 (32.8)	28 (24.8)	^a^	19 (21.8)	^a^	^a^	^a^	10 (33.3)	5 (11.1)
	Type 2	503 (74.6)	147 (74.6)	44 (83.0)	77 (66.4)	83 (73.5)	8 (80.0)	68 (78.2)	^a^	15 (83.3)	^a^	19 (63.3)	38 (84.4)
	Other type	11 (1.6)	5 (2.5)	0 (0.0)	^a^	^a^	^a^	0 (0.0)	^a^	0 (0.0)	^a^	^a^	^a^
Age	Years, median (IQR)	72 (60–81)	67 (58–79)	72 (59–84)	75 (59–83)	70 (61–78)	70 (53–78)	76 (64–82)	78 (55–89)	86 (82–89)	87 (87–87)	72 (42–77)	77 (67–86)
CCI, median (IQR)	Score, median (IQR)	6 (4–7)	5 (4–8)	6 (4–8)	6 (4–7)	5 (3–7)	4 (2–6)	7 (4–8)	6 (4–6)	7 (6–9)	8 (8–8)	6 (2–9)	6 (4–8)

Abbreviations: CCI, Charlson Comorbidity Index; IQR, interquartile range.

^a^ Small data suppression has been carried out for all values where *n* < 5 and is populated.

^b^ Excluding unknown not listed on hospital records.

The median age was 72 years (IQR 60–81), with a male‐to‐female ratio of 1.2:1. The median CCI was 6 (IQR 4–7). Among the included people, 160 (23.7%) had type 1 diabetes, 503 (74.6%) had type 2 diabetes, and 11 (1.6%) had other forms of diabetes, including type 3c, gestational or iatrogenic. Ethnicity data were available for 613 people, of whom 71.5% were White, 19.2% Asian, 7.5% Black and 1.8% Mixed/Other.

Insulin use was recorded in 934 (77.5%) episodes. Biguanides were prescribed in 361 (30.0%) episodes, dipeptidyl peptidase‐4 inhibitors in 159 (13.2%), sulfonylureas in 107 (8.9%), sodium‐glucose transport protein 2 inhibitors in 82 (6.8%), glucagon‐like peptide 1 agonists in 14 (1.2%), and thiazolidinediones in 4 (0.3%) (Table 2). Antipsychotic use was identified in 81 (6.7%) episodes. Hypoglycaemia with cognitive impairment was present in 356 (49.5%) cases, with 309 requiring external support (and subsequent classification as a Level 3 event).

**TABLE 2:** Data on episodes—all results by hospital. All results given as *n* (%), where *n* represents the number of episodes.

	Centre	Overall	A	B	C	D	E	F	G	H	I	J	K
Overall cohort		1205 (100.0)	356 (29.5)	107 (8.9)	333 (27.6)	144 (12.0)	10 (0.8)	90 (7.5)	10 (0.8)	41 (3.4)	^a^	47 (3.9)	63 (5.2)
Sex	Female	645 (53.5)	178 (50.0)	64 (59.8)	201 (60.4)	86 (59.7)	^a^	40 (44.4)	9 (90.0)	11 (26.8)	^a^	15 (31.9)	38 (60.3)
	Male	560 (46.5)	178 (50.0)	43 (40.2)	132 (39.6)	58 (40.3)	7 (70.0)	50 (55.6)	^a^	30 (73.2)	^a^	32 (68.1)	25 (39.7)
Ethnicity^b^	White	777 (70.1)	228 (65.5)	17 (17.9)	269 (84.6)	90 (68.2)	8 (80.0)	68 (87.2)	^a^	19 (79.2)	^a^	32 (80.0)	42 (85.7)
	Asian	201 (18.1)	77 (22.1)	58 (61.1)	9 (2.8)	26 (19.7)	^a^	6 (7.7)	5 (50.0)	^a^	^a^	5 (12.5)	7 (14.3)
	Black	116 (10.5)	39 (11.2)	11 (11.6)	40 (12.6)	15 (11.4)	^a^	^a^	^a^	^a^	^a^	^a^	^a^
	Other/mixed	14 (1.3)	^a^	9 (9.5)	0 (0.0)	^a^	0 (0.0)	0 (0.0)	0 (0.0)	0 (0.0)	^a^	0 (0.0)	0 (0.0)
Diabetes	Type 1	358 (29.7)	113 (31.7)	14 (13.1)	144 (43.2)	41 (28.5)	^a^	19 (21.1)	^a^	5 (12.2)	^a^	11 (23.4)	8 (12.7)
	Type 2	829 (68.8)	237 (66.6)	93 (86.9)	188 (56.5)	98 (68.1)	8 (80.0)	71 (78.9)	9 (90.0)	36 (87.8)	^a^	32 (68.1)	53 (84.1)
	Other type	18 (1.5)	6 (1.7)	0 (0.0)	^a^	5 (3.5)	0 (0.0)	0 (0.0)	0 (0.0)	0 (0.0)	^a^	^a^	^a^
Antidiabetic medications	Insulin	934 (77.5)	269 (75.6)	82 (76.6)	273 (82.0)	97 (67.4)	8 (80.0)	69 (76.7)	7 (70.0)	39 (95.1)	^a^	42 (89.4)	44 (69.8)
	Biguanides	361 (30.0)	97 (27.2)	43 (40.2)	83 (24.9)	43 (29.9)	^a^	33 (36.7)	0 (0.0)	16 (39.0)	^a^	20 (42.6)	20 (31.7)
	Sulfonylureas	107 (8.9)	25 (7.0)	27 (25.2)	18 (5.4)	13 (9.0)	^a^	15 (16.7)	0 (0.0)	^a^	^a^	^a^	^a^
	DPP4I	159 (13.2)	44 (12.4)	18 (16.8)	50 (15.0)	18 (12.5)	^a^	13 (14.4)	0 (0.0)	^a^	^a^	^a^	12 (19.0)
	SGLT2	82 (6.8)	21 (5.9)	19 (17.8)	10 (3.0)	5 (3.5)	^a^	6 (6.7)	0 (0.0)	^a^	^a^	12 (25.5)	9 (14.3)
	GLP‐1	14 (1.2)	^a^	6 (5.6)	^a^	^a^	^a^	^a^	0 (0.0)	^a^	^a^	^a^	^a^
	Thiazolidinediones	^a^	^a^	^a^	^a^	^a^	^a^	^a^	0 (0.0)	^a^	^a^	^a^	^a^
	Antipsychotics	81 (6.7)	17 (4.8)	^a^	47 (14.1)	^a^	^a^	6 (6.7)	0 (0.0)	^a^	^a^	^a^	^a^
Event level	2	896 (74.4)	260 (73.0)	92 (86.0)	266 (79.9)	86 (59.7)	6 (60.0)	39 (43.3)	9 (90.0)	36 (87.8)	^a^	44 (93.6)	56 (88.9)
	3	309 (25.6)	96 (27.0)	15 (14.0)	67 (20.1)	58 (40.3)	^a^	51 (56.7)	^a^	5 (12.2)	^a^	^a^	7 (11.1)
Cognitive impairment^b^	Yes	356 (49.5)	145 (57.5)	13 (38.2)	55 (30.9)	68 (64.8)	^a^	44 (57.9)	^a^	10 (52.6)	^a^	^a^	13 (44.8)
	No	363 (50.5)	107 (42.5)	21 (61.8)	123 (69.1)	37 (35.2)	^a^	32 (42.1)	^a^	9 (47.4)	^a^	14 (87.5)	16 (55.2)
Precipitant	Fasting/missed meals	490 (40.7)	201 (56.5)	73 (68.2)	131 (39.3)	32 (22.2)	^a^	17 (18.9)	6 (60.0)	17 (41.5)	^a^	^a^	9 (14.3)
	Intercurrent illness	539 (44.7)	112 (31.5)	77 (72.0)	175 (52.6)	96 (66.7)	5 (50.0)	16 (17.8)	10 (100.0)	^a^	^a^	^a^	45 (71.4)
	Incorrect medication	201 (16.7)	29 (8.1)	9 (8.4)	105 (31.5)	11 (7.6)	^a^	39 (43.3)	^a^	^a^	^a^	^a^	^a^
	Exercise	^a^	0 (0.0)	0 (0.0)	^a^	0 (0.0)	^a^	0 (0.0)	^a^	^a^	^a^	^a^	^a^
	Unclear	298 (24.7)	78 (21.9)	11 (10.3)	78 (23.4)	28 (19.4)	^a^	26 (28.9)	^a^	20 (48.8)	^a^	44 (93.6)	7 (11.1)
	Multiple factors	174 (14.4)	57 (16.0)	6 (5.6)	92 (27.6)	18 (12.5)	^a^	0 (0.0)	^a^	^a^	^a^	^a^	^a^
	Single factor	733 (60.8)	221 (62.1)	90 (84.1)	163 (48.9)	98 (68.1)	8 (80.0)	64 (71.1)	10 (100.0)	20 (48.8)	^a^	^a^	56 (88.9)
Furthest line treatment reached	Not fully documented	306 (25.4)	49 (13.8)	27 (25.2)	106 (31.8)	26 (18.1)	^a^	14 (15.6)	^a^	5 (12.2)	^a^	25 (53.2)	48 (76.2)
	Oral glucose	389 (32.3)	97 (27.2)	54 (50.5)	140 (42.0)	23 (16.0)	^a^	31 (34.4)	^a^	17 (41.5)	^a^	15 (31.9)	7 (11.1)
	IV Dextrose	460 (38.2)	196 (55.1)	23 (21.5)	79 (23.7)	85 (59.0)	6 (60.0)	38 (42.2)	^a^	17 (41.5)	^a^	7 (14.9)	^a^
	Glucagon	49 (4.1)	14 (3.9)	^a^	8 (2.4)	10 (6.9)	^a^	7 (7.8)	^a^	^a^	^a^	0 (0.0)	^a^
Total number of episodes where treatment has been used	Oral glucose	631 (70.7)	209 (73.3)	68 (87.2)	177 (72.8)	61 (55.0)	^a^	49 (55.1)	^a^	31 (88.6)	^a^	20 (95.2)	9 (64.3)
	IV Dextrose	494 (43.4)	208 (58.6)	23 (25.3)	84 (25.2)	95 (66.0)	7 (77.8)	43 (48.3)	^a^	17 (41.5)	^a^	7 (28.0)	5 (12.2)
	Glucagon	49 (3.0)	14 (3.0)	^a^	8 (1.5)	10 (5.8)	^a^	7 (5.5)	^a^	^a^	^a^	0 (0.0)	^a^
Hyperglycaemia	Yes	27 (2.2)	5 (1.4)	^a^	10 (3.0)	7 (4.9)	0 (0.0)	0 (0.0)	0 (0.0)	0 (0.0)	^a^	^a^	^a^
	No	1178 (97.8)	351 (98.6)	105 (98.1)	323 (97.0)	137 (95.1)	10 (100.0)	90 (100.0)	10 (100.0)	41 (100.0)	^a^	45 (95.7)	62 (98.4)
Episode outcome	Care continued as an inpatient	1115 (92.5)	332 (93.3)	100 (93.5)	328 (98.5)	120 (83.3)	9 (90.0)	69 (76.7)	10 (100.0)	41 (100.0)	^a^	46 (97.9)	56 (88.9)
	Sent to ITU	12 (1.0)	6 (1.7)	0 (0.0)	0 (0.0)	^a^	0 (0.0)	^a^	0 (0.0)	0 (0.0)	^a^	0 (0.0)	0 (0.0)
	Sent home	5 (0.4)	0 (0.0)	^a^	0 (0.0)	^a^	0 (0.0)	^a^	0 (0.0)	0 (0.0)	^a^	0 (0.0)	0 (0.0)
	Death	73 (6.1)	18 (5.1)	5 (4.7)	5 (1.5)	19 (13.2)	^a^	17 (18.9)	0 (0.0)	0 (0.0)	^a^	^a^	7 (11.1)
Length of stay from episode start	Days, Median (IQR)	8 (3–19)	9 (3–19)	6 (3–16)	8 (4–21)	14 (4–34)	1 (0–5)	6 (2–15)	27 (4–59)	14 (5–28)	9 (6–9)	9 (4–16)	5 (2–17)
Discharge outcome	Died	273 (22.7)	87 (24.4)	19 (17.8)	85 (25.5)	51 (35.4)	^a^	17 (18.9)	0 (0.0)	0 (0.0)	^a^	^a^	9 (14.3)
	Complex	469 (38.9)	162 (45.5)	19 (17.8)	120 (36.0)	51 (35.4)	^a^	31 (34.4)	^a^	28 (68.3)	^a^	20 (42.6)	32 (50.8)
	Simple	373 (31.0)	86 (24.2)	56 (52.3)	93 (27.9)	31 (21.5)	5 (50.0)	41 (45.6)	5 (50.0)	11 (26.8)	^a^	21 (44.7)	20 (31.7)
	Other	90 (7.5)	21 (5.9)	13 (12.1)	35 (10.5)	11 (7.6)	0 (0.0)	^a^	^a^	^a^	^a^	^a^	^a^
Adjustment	Medication change	580 (48.1)	125 (35.1)	73 (68.2)	161 (48.3)	71 (49.3)	5 (50.0)	55 (61.1)	10 (100.0)	24 (58.5)	^a^	18 (38.3)	34 (54.0)
	Specialist team	340 (28.2)	62 (17.4)	51 (47.7)	115 (34.5)	44 (30.6)	8 (80.0)	15 (16.7)	7 (70.0)	^a^	^a^	8 (17.0)	29 (46.0)
	Palliative care	210 (17.4)	86 (24.2)	34 (31.8)	34 (10.2)	30 (20.8)	0 (0.0)	9 (10.0)	0 (0.0)	0 (0.0)	^a^	6 (12.8)	11 (17.5)
	CGM started	26 (2.2)	10 (2.8)	^a^	6 (1.8)	^a^	^a^	^a^	0 (0.0)		^a^	0 (0.0)	
	Glucagon prescribed	^a^	^a^	0 (0.0)	^a^	0 (0.0)	0 (0.0)	^a^	0 (0.0)	0 (0.0)	^a^	0 (0.0)	0 (0.0)
Medication change, % of all medication changes	Stop sulfonylureas	40 (6.9)	5 (4.0)	9 (12.3)	5 (3.1)	8 (11.3)	0 (0.0)	6 (10.9)	0 (0.0)	0 (0.0)	^a^	^a^	^a^
	Reduce sulfonylureas	24 (4.1)	6 (4.8)	9 (12.3)	^a^	^a^	0 (0.0)	^a^	0 (0.0)	0 (0.0)	^a^	0 (0.0)	^a^
	Stop insulin	85 (14.7)	15 (12.0)	^a^	36 (22.4)	17 (23.9)	0 (0.0)	5 (9.1)	^a^	^a^	^a^	^a^	^a^
	Reduce insulin	414 (71.4)	96 (76.8)	50 (68.5)	113 (70.2)	39 (54.9)	5 (100.0)	43 (78.2)	9 (90.0)	22 (91.7)	^a^	8 (44.4)	25 (73.5)
	Changes to others	111 (19.1)	24 (19.2)	14 (19.2)	18 (11.2)	16 (22.5)	^a^	9 (16.4)	0 (0.0)	5 (20.8)	^a^	10 (55.6)	14 (41.2)
Recurrent episodes by admission	First episodes	741 (61.5)	227 (63.8)	51 (47.7)	142 (42.6)	110 (76.4)	10 (100.0)	88 (97.8)	6 (60.0)	22 (53.7)	^a^	34 (72.3)	50 (79.4)
	Recurrent episode occurring in same admission as first	464 (38.5)	129 (36.2)	56 (52.3)	191 (57.4)	34 (23.6)	0 (0.0)	^a^	^a^	19 (46.3)	^a^	13 (27.7)	13 (20.6)

Abbreviations: CCI, Charlson Comorbidity Index; CGM, continuous glucose monitoring; DDP4I, dipeptidyl peptidase‐4 inhibitors; GLP‐1, glucagon‐like peptide‐1mimetic; IQR, interquartile range; ITU, intensive treatment unit; IV, intravenous; SGLT2, sodium‐glucose cotransporter‐2 inhibitor.

^a^ Small data suppression has been carried out for all values, where *n* < 5 and is populated.

^b^ Excluding unknown not listed on hospital records.

Precipitating factors were identified in 733 (60.8%) episodes, with intercurrent illness being the most common (44.7%), followed by fasting/missed meals (40.7%), incorrect medication dosage (16.7%) and exercise (0.1%). No clear precipitant was identified in 24.7% of cases, and multiple precipitants were noted in 14.4% (Table S1).

### 3.1 Adherence to JBDS hypoglycaemia management guidelines

The treatment modalities used to resolve hypoglycaemia varied. Intravenous dextrose was used in 460 (38.2%) episodes, followed by oral glucose in 389 (32.3%), and intramuscular glucagon in 49 (4.1%). Of the 460 episodes where intravenous dextrose was used, there were 31 (6.7%) instances where a 50% preparation of intravenous dextrose was administered. In 306 (25.4%) episodes, the furthest line of treatment according to the JBDS guidelines—the most escalated intervention required during an episode, stepwise from oral glucose, intravenous dextrose and intramuscular glucagon—was not documented (Table 2).

Differences in treatment approaches were observed between IHSG Levels 2 and 3 episodes (Table 3). Level 2 episodes were more likely to be managed with oral glucose (Level 2 vs. Level 3: 38.4% vs. 14.6%, *p* < 0.0001), whereas Level 3 episodes more frequently required intravenous dextrose (Level 2 vs. Level 3: 28.9% vs. 65%, *p* < 0.0001) or glucagon (Level 2 vs. Level 3: 1.5% vs. 11.7%, *p* < 0.0001). Documentation of the furthest intervention was also more frequently incomplete in Level 2 episodes (Level 2 vs. Level 3: 31.1% vs. 8.7%, *p* < 0.0001).

**TABLE 3:** Data on episodes—all results by IHSG level. All results given as *n* (%), where *n* represents the number of episodes.

	IHSG level	Level 2	Level 3	*p*
Cohort		896 (74.4)	309 (25.6)	
Sex	Female	509 (56.8)	136 (44.0)	0.0001
	Male	387 (43.2)	173 (56.0)	
	Ratio F:M	1:1.315	1.272:1	
Ethnicity^a^	White	566 (68.7)	211 (74.3)	
	Asian	153 (18.6)	48 (16.9)	
	Black	93 (11.3)	23 (8.1)	
	Other/mixed	12 (1.5)	2 (0.7)	
Diabetes	Type 1	287 (32.0)	71 (23.0)	0.0007
	Type 2	591 (66.0)	238 (77.0)	
	Other	18 (2.0)	0 (0.0)	
Age	Median (IQR)	74 (58–83)	70 (60–79)	0.0167
CCI	Median (IQR)	6 (4–7)	6 (4–8)	
Antidiabetic medications	Insulin	715 (79.8)	219 (70.9)	0.0015
	Biguanides	279 (31.1)	82 (26.5)	
	Sulfonylureas	79 (8.8)	28 (9.1)	
	DPP4I	100 (11.2)	59 (19.1)	0.0006
	SGLT2	67 (7.5)	15 (4.9)	
	GLP‐1	11 (1.2)	3 (1.0)	
	Thiazolidinediones	3 (0.3)	1 (0.3)	
	Antipsychotics	54 (6.0)	27 (8.7)	
Cognitive impairment^a^	Yes	98 (21.4)	309 (100.0)	<0.0001
	No	359 (78.6)	0 (0.0)	
Precipitant	Fasting/missed meals	366 (40.8)	124 (40.1)	
	Intercurrent illness	385 (43.0)	154 (49.8)	0.0397
	Incorrect medication	146 (16.3)	55 (17.8)	
	Exercise	1 (0.1)	0 (0.0)	
	Unclear	231 (25.8)	67 (21.7)	
	Multiple	128 (14.3)	47 (15.2)	
	Single	768 (85.7)	262 (84.8)	
Furthest line treatment reached	Not fully documented	279 (31.1)	27 (8.7)	<0.0001
	Oral glucose	344 (38.4)	45 (14.6)	<0.0001
	IV Dextrose	259 (28.9)	201 (65.0)	<0.0001
	Glucagon	13 (1.5)	36 (11.7)	<0.0001
Total number of episodes treatment used^a^	Oral glucose	485 (76.3)	146 (57.0)	<0.0001
	IV dextrose	267 (29.8)	227 (73.5)	<0.0001
	Glucagon	13 (1.7)	36 (12.3)	<0.0001
Hyperglycaemia	Yes	17 (1.9)	10 (3.2)	
	No	879 (98.1)	299 (96.8)	
Episode outcome	Care continued as inpatient	865 (96.5)	250 (80.9)	<0.0001
	Sent to ITU	1 (0.1)	11 (3.6)	<0.0001
	Sent home	4 (0.4)	1 (0.3)	
	Death	26 (2.9)	47 (15.2)	<0.0001
Length of stay after hypoglycaemia start	Days, median (IQR)	8 (3–20)	9 (3–19)	
Discharge outcome	Died	145 (16.2)	128 (41.4)	<0.0001
	Complex	374 (41.7)	95 (30.7)	0.0007
	Simple	308 (34.4)	65 (21.0)	<0.0001
	Other	69 (7.7)	21 (6.8)	
Adjustment	Medication change	421 (47.0)	159 (51.5)	
	Specialist team	237 (26.5)	103 (33.3)	0.023
	Palliative care	136 (15.2)	74 (23.9)	0.0007
	CGM started	17 (1.9)	9 (2.9)	
	Glucagon prescribed	1 (0.1)	2 (0.6)	
Medication change, % of all medication changes	Stop sulfonylureas	29 (6.9)	11 (6.9)	
	Reduce sulfonylureas	22 (5.2)	2 (1.3)	0.0343
	Stop insulin	51 (12.1)	34 (21.4)	0.0081
	Reduce insulin	307 (72.9)	107 (67.3)	
	Changes to others	77 (18.3)	34 (21.4)	
Recurrent episodes by admission	First episodes	527 (58.8)	214 (69.3)	0.0011
	Recurrent episode occurring in same admission as first	369 (41.2)	95 (30.7)	

Abbreviations: CCI, Charlson Comorbidity Index; CGM, continuous glucose monitoring; DDP4I, dipeptidyl peptidase‐4 inhibitors; GLP‐1, glucagon‐like peptide‐1mimetic; IQR, interquartile range; ITU, intensive treatment unit; IV, intravenous; SGLT2, sodium‐glucose cotransporter‐2 inhibitor.

^a^ Excluding unknowns not listed on hospital records.

There was marked variation in management practices of hypoglycaemic episodes between centres, including treatment approach, length of stay and post‐hypoglycaemia medication adjustments (Table 2; Table S1). A direct comparison has been made between Centres A and D in this study for illustrative purposes. Notably, Centre A utilised oral glucose more frequently compared to Centre D (Centre A vs. Centre D, 27.2% vs. 16.0%, *p* = 0.0077) and had lower rates of post‐treatment hyperglycaemia (Centre A vs. Centre D, 1.4% vs. 4.9%, *p* = 0.0455), whereas Centre D had a longer median length of stay (Centre A vs. Centre D, 9 (3–19) days vs. 14 (4–34) days, *p* = 0.0286), and more medication changes as a result of hypoglycaemia (Centre A vs. Centre D, 35.1% vs. 49.3%, *p* = 0.0045) (Table S2).

### 3.2 Applicability of IHSG classifications in hospitalised people

Of the 1205 episodes, 309 (25.6%) were classified as IHSG Level 3 and 896 (74.4%) as Level 2 (Table 3; Table S3). Level 3 hypoglycaemia was associated with increased mortality compared to level 2 episodes (Level 3 vs. Level 2, 15.2% vs. 2.9%, *p* < 0.0001). Level 3 episodes also led to higher rates of ITU escalation (Level 3 vs. Level 2, 3.6% vs. 0.1%, *p* < 0.0001), specialist input including specialist nurses, registrars or consultants in diabetes (Level 3 vs. Level 2, 33.3% vs. 26.5%, *p* = 0.023) or palliative care input (Level 3 vs. Level 2, 23.9% vs. 15.2%, *p* = 0.0007). The observed outcomes reflect associations rather than causation.

### 3.3 Gaps in care processes amenable to quality improvement interventions

There were 464 (38.5%) recurrent episodes that occurred in the same admission as the first episode (Table S4). Recurrence was more common in women (304 women; 65.5% vs. 34.5%, *p* < 0.0001) and in people from Afro‐Caribbean ethnicity (55 people; 12.8%, *p* = 0.0444) compared to men and other ethnic groups, respectively. A greater proportion of the recurrent episodes occurred in people with type 1 diabetes (35.6% of the recurrent episodes), compared to the proportion of the first episodes (26% of the first episodes). Subsequently, a reduced proportion of the recurrent episodes occurred in people with type 2 diabetes (63.1% of the recurrent episodes), compared to the proportion of the first episodes (72.3% of the first episodes). Recurrence was also more frequent among those receiving insulin therapy (proportion of recurrent vs. initial episodes, 83.4% vs. 73.8%, *p* < 0.0001) or antipsychotic medications (proportion of recurrent vs. initial episodes, 9.5% vs. 5%, *p* = 0.003).

Compared to the first episodes, recurrent events were more commonly precipitated by fasting (proportion of recurrent episodes vs. first episodes 48.1% vs. 36.0%, *p* < 0.0001), intercurrent illness (proportion of recurrent episodes vs. first episodes 48.5% vs. 42.4%, *p* = 0.0429) and incorrect medication dosing (proportion of recurrent episodes vs. first episodes 19.6% vs. 14.8%, *p* = 0.0323). Oral glucose was more frequently used for recurrent events compared to first events (37.5% vs. 29%, *p* = 0.0024). These recurrent episodes were associated with longer hospital stays (median 10 (5–24) days, *p* < 0.0001) compared to first episodes (median 7 (3–18) days, *p* < 0.0001) and complex discharges (recurrent episode vs. first episode, 45% vs. 35.1%, *p* = 0.0007). Notably, first episodes were more likely to be associated with Level 3 hypoglycaemic episodes (28.9%) and to result in mortality (8.0%; *p* = 0.0004), as opposed to recurrent episodes (3.0%; *p* = 0.0004).

Eighty‐three (11.2%) people were re‐admitted within the study timeframe with another hypoglycaemic episode (Table S5). These recurrent admissions resulted in fewer deaths (8.4%, *p* = 0.0016), in contrast to first admissions (23.2%, *p* = 0.0016), but higher rates of complex discharge (50.6%, *p* = 0.0021), in contrast to first admission (32.8%, p = 0.0021), suggesting an unmet need for follow‐up and transitional care.

In terms of therapeutic adjustments, medication changes were made in 580 (48.1%) episodes, most commonly insulin dose reductions (71.4% of changes), followed by adjustments to other oral diabetes medications (30.1%) and insulin cessation (14.7%; Table 2; Table S1). Specialist team involvement occurred in 28.2% of episodes, palliative care in 17.4%, CGM initiation in 2.2% and glucagon prescriptions in 0.2%.

Marked centre‐level differences were also identified in episode demographics, precipitants, treatment modalities and outcomes (Table S2). Centre D exhibited a higher rate of patient deaths post‐episode (13.2%, *p* = 0.0039) and longer lengths of stay (14 (4–34), *p* = 0.0286), whereas Centre A had higher rates of complex discharges (45.5%, *p* = 0.0457) and better documentation. These inter‐centre discrepancies further underscore the need for standardised care protocols and quality improvement initiatives.

## 4 DISCUSSION

Despite JBDS guidance, this multicentre study found significant variation in how clinically significant hypoglycaemia is managed across UK hospitals, underscoring inconsistent adherence to best practices and the need for a coordinated national strategy and surveillance system. Severe hypoglycaemia occurred most often in older adults with type 2 diabetes, especially those with multimorbidity and from ethnic minority backgrounds—supporting emerging evidence that the risks in this group remain underestimated.^19^, ^20^

More than 75% of episodes were linked to potentially modifiable or contributory factors, although some, such as intercurrent illness, may not be directly modifiable. These were most commonly fasting or missed meals, particularly around illness or procedures, emphasising the need for meal protection and medication adjustment.^21^ Yet, glucagon was underutilised both during episodes and in discharge planning, consistent with longstanding concerns about its underuse in type 2 diabetes.^22^ Similarly, CGM initiation was rare, though emerging evidence suggests potential value in high‐risk inpatients.^23^

Only 28.2% of cases were referred to diabetes teams, despite half of the episodes being associated with cognitive impairment. Death occurred in 6.1% of cases, demonstrating an association between clinically significant hypoglycaemia and poor outcomes. Studies have shown that cognitive impairment is associated with hypoglycaemia. However, we would not be able to conclude the direction of this association through our data.

Recurrent episodes—more common in women, people from Afro‐Caribbean ethnicity, and those on insulin or with type 1 diabetes—were often triggered by similar causes and required less intensive intervention, suggesting earlier detection but also an ongoing failure to fully address preventable risks when the cause is potentially modifiable. Patient education remains vital.^24^

Type 2 diabetes was more often associated with severe hypoglycaemia and worse outcomes than type 1, consistent with demographic and clinical trends.^25^ Sex‐based differences also emerged: women had more frequent and recurrent episodes; men had more isolated, severe events requiring IM glucagon—findings echoed in previous studies.^26^, ^27^

Finally, wide variation between hospitals in treatment and outcomes—even between similar centres—highlights the need for national benchmarking tools like the DEKODE model to standardise care and drive improvement at scale.

A key strength of this study is its status as the largest real‐world analysis in the United Kingdom to stratify inpatient hypoglycaemic events using the IHSG classification and examine associated clinical outcomes. The scale and real‐world nature of the study enhance generalisability and provide valuable insight into current inpatient diabetes care. Further, since all participating hospitals have an electronic record of all glucose readings, the automated glucose report generation by the hospital's health informatics team, combined with manual verification of clinically significant hypoglycaemia, reduces the risk of underreporting in the DEKODE model. The use of the IHSG classification allowed severity‐based stratification, supporting its clinical utility. However, the retrospective design risks relying on potential incomplete documentation of the episode, particularly around CGM use, referrals and treatment rationale, which may limit interpretability. We are also unsure if hypoglycaemia detected through CGM was then verified through capillary glucose testing. Furthermore, this system relies on healthcare workers and is susceptible to recording bias. Other factors, such as the accuracy of data entry during data collection and the generation of episode lists from POC (point‐of‐care) testing data, can also affect data quality.

High mortality may reflect underlying frailty and multimorbidity rather than hypoglycaemia as a direct cause. However, we are not able to confirm this from our current data. Prospective studies are needed to explore the causal contribution of hypoglycaemia to adverse outcomes, particularly in older, multimorbid populations with type 2 diabetes. There is also variability in intercurrent illness/sepsis, and it may not be a modifiable risk factor in all cases. Evaluating the utility and cost‐effectiveness of inpatient CGM, especially in high‐risk groups, is another key area. Further studies on how the adoption of CGM, both inpatient and outpatient, alongside patient education and structured follow‐up, could reduce the risk of hypoglycaemia across diabetes types is needed. We also did not explore other forms of anti‐diabetes medicines and their association with hypoglycaemia in this study and propose further studies in the future on this.

Additionally, qualitative research should explore barriers to glucagon use and disparities in care by sex and ethnicity to inform equitable, patient‐centred approaches. Ultimately, implementation‐focused research will be essential to embed best practices and reduce avoidable harm from inpatient hypoglycaemia. Despite these limitations, the study provides robust, actionable evidence to inform more consistent, proactive inpatient hypoglycaemia care across the NHS.

## 5 CONCLUSIONS

This study establishes the framework for a robust national dataset for clinically significant hypoglycaemia in those admitted to hospital, validating IHSG classification in practice and emphasising the high mortality and morbidity associated with these events. Improved adherence to care standards, targeted risk reduction strategies and systematic auditing are crucial to preventing avoidable harm and ensuring equitable, high‐quality inpatient diabetes care across the NHS.

## AUTHOR CONTRIBUTIONS

CP and MI have contributed equally to all aspects of this study and are joint first authors. PK conceptualised the study and refined the design and methodology in collaboration with all authors. AB, AK, AL, AR, CP, KP, MI and SSM contributed to data curation, investigation, management and methodology. AB, CP and MI oversaw project administration. PK provided the resources needed for the study and supervised the project. AB, AK, AL, CP, KP, MI and SSM supervised local teams in participating centres. AI, AS, LR, PC, AM, PK and RR all provided senior supervision and project administration. SSM analysed the data and helped with visualisation. The DEVI collaborative group contributed to methodology, data collection, project administration, resources, software and supervision. All authors were involved in writing the article (original draft and review/editing).

## FUNDING INFORMATION

This work was not directly funded. PK is supported by the National Institute for Health and Care Research (NIHR) through his Advanced Fellowship (NIHR303671). Additionally, he is supported by the Midlands Patient Safety Research Collaboration (PSRC) and the NIHR Race, Equity and Diversity in Careers Incubator. The views expressed in this study are those of the author and do not necessarily reflect the official positions of the NIHR or the Department of Health and Social Care.

## CONFLICT OF INTEREST STATEMENT

None to declare.

## ETHICS STATEMENT

This study was approved as part of service improvement by the Departments of Information Governance of University Hospitals Birmingham NHS Foundation Trust (CARMS approval number: 20766), the Information Governance Team of Sandwell and West Birmingham Hospitals NHS Trust (Audit ID: 2555), the Information Governance Team of Walsall Manor Hospital (Audit reference ID: QP24‐73), the Information Governance Team of Russells Hall Hospital (Audit reference: AUDIT DEKODE) and the Information Governance Team of Royal Wolverhampton NHS Trust (Audit ID: 2765).

## Supporting information


**Table S1.** Data on episodes—all results by hospital. All results given as *n* (%), where *n* represents the number of episodes.
Table S2. Data on episodes—results between two similar centres A and D. All results given as *n* (%), where *n* represents the number of episodes.
Table S3. Baseline data on episodes—all results by IHSG level. All results given as *n* (%), where *n* represents the number of episodes.
Table S4. Data on episodes—all results by episode type (first episode or recurrent). All results given as *n* (%), where *n* represents the number of episodes.
Table S5. Data on admission—all results by admission type (first admission or recurrent admissions). All results given as *n* (%), where *n* represents the number of admissions.

## Data Availability

The datasets are available from the corresponding author upon reasonable request. PK is the guarantor of this work, having had full access to all the data in the study and takes responsibility for the integrity of the data.
