# Supplementary material for: Clinically significant hypoglycaemia in hospitalised adults: A multicentre study of 1205 episodes in the United Kingdom
Source: Diabet Med. 2026 Apr 20;43(6):e70331. doi: 10.1111/dme.70331 (PMC13170989; doi:10.1111/dme.70331)
Supplement: Supplementary file 1 — Table S1. Data on episodes—all results by hospital. All results given as n (%), where n represents the number of episodes. Table S2. Data on episodes—results between two similar centres A and D. All results given as n (%), where n represents the number of episodes. Table S3. Baseline data on episodes—all results by IHSG level. All results given as n (%), where n represents the number of episodes. Table S4. Data on episodes—all results by episode type (first episode or recurrent). All results given as n (%), where n represents the number of episodes. Table S5. Data on admission—all results by admission type (first admission or recurrent admissions). All results given as n (%), where n represents the number of admissions. [file DME-43-e70331-s001.docx]

Supplementary Table 1: Data on episodes - all results by hospital. All results given as n (%), where n represents the number of episodes.

|  | Centre | Overall | A | B | C | D | E | F | G | H | I | J | K |
| --- | --- | --- | --- | --- | --- | --- | --- | --- | --- | --- | --- | --- | --- |
| Number of episodes (%) |  | 1205 (100.0) | 356 (29.5) | 107 (8.9) | 333 (27.6) | 144 (12.0) | 10 (0.8) | 90 (7.5) | 10 (0.8) | 41 (3.4) | * | 47 (3.9) | 63 (5.2) |
| Sex | Female | 645 (53.5) | 178 (50.0) | 64 (59.8) | 201 (60.4) | 86 (59.7) | * | 40 (44.4) | 9 (90.0) | 11 (26.8) | * | 15 (31.9) | 38 (60.3) |
|  | Male | 560 (46.5) | 178 (50.0) | 43 (40.2) | 132 (39.6) | 58 (40.3) | 7 (70.0) | 50 (55.6) | * | 30 (73.2) | * | 32 (68.1) | 25 (39.7) |
| Ethnicity** | White | 777 (70.1) | 228 (65.5) | 17 (17.9) | 269 (84.6) | 90 (68.2) | 8 (80.0) | 68 (87.2) | * | 19 (79.2) | * | 32 (80.0) | 42 (85.7) |
|  | Asian | 201 (18.1) | 77 (22.1) | 58 (61.1) | 9 (2.8) | 26 (19.7) | * | 6 (7.7) | 5 (50.0) | * | * | 5 (12.5) | 7 (14.3) |
|  | Black | 116 (10.5) | 39 (11.2) | 11 (11.6) | 40 (12.6) | 15 (11.4) | * | * | * | * | * | * | * |
|  | Other/mixed | 14 (1.3) | * | 9 (9.5) | 0 (0.0) | * | 0 (0.0) | 0 (0.0) | 0 (0.0) | 0 (0.0) | * | 0 (0.0) | 0 (0.0) |
| Diabetes | Type 1 | 358 (29.7) | 113 (31.7) | 14 (13.1) | 144 (43.2) | 41 (28.5) | * | 19 (21.1) | * | 5 (12.2) | * | 11 (23.4) | 8 (12.7) |
|  | Type 2 | 829 (68.8) | 237 (66.6) | 93 (86.9) | 188 (56.5) | 98 (68.1) | 8 (80.0) | 71 (78.9) | 9 (90.0) | 36 (87.8) | * | 32 (68.1) | 53 (84.1) |
|  | Other type | 18 (1.5) | 6 (1.7) | 0 (0.0) | * | 5 (3.5) | 0 (0.0) | 0 (0.0) | 0 (0.0) | 0 (0.0) | * | * | * |
| Event level | 2 | 896 (74.4) | 260 (73.0) | 92 (86.0) | 266 (79.9) | 86 (59.7) | 6 (60.0) | 39 (43.3) | 9 (90.0) | 36 (87.8) | * | 44 (93.6) | 56 (88.9) |
|  | 3 | 309 (25.6) | 96 (27.0) | 15 (14.0) | 67 (20.1) | 58 (40.3) | * | 51 (56.7) | * | 5 (12.2) | * | * | 7 (11.1) |
| Precipitant | Fasting/missed meals | 490 (40.7) | 201 (56.5) | 73 (68.2) | 131 (39.3) | 32 (22.2) | * | 17 (18.9) | 6 (60.0) | 17 (41.5) | * | * | 9 (14.3) |
|  | Intercurrent illness | 539 (44.7) | 112 (31.5) | 77 (72.0) | 175 (52.6) | 96 (66.7) | 5 (50.0) | 16 (17.8) | 10 (100.0) | * | * | * | 45 (71.4) |
|  | Incorrect medication | 201 (16.7) | 29 (8.1) | 9 (8.4) | 105 (31.5) | 11 (7.6) | * | 39 (43.3) | * | * | * | * | * |
|  | Exercise | * | 0 (0.0) | 0 (0.0) | * | 0 (0.0) | * | 0 (0.0) | * | * | * | * | * |
|  | Unclear | 298 (24.7) | 78 (21.9) | 11 (10.3) | 78 (23.4) | 28 (19.4) | * | 26 (28.9) | * | 20 (48.8) | * | 44 (93.6) | 7 (11.1) |
|  | Multiple factors | 174 (14.4) | 57 (16.0) | 6 (5.6) | 92 (27.6) | 18 (12.5) | * | 0 (0.0) | * | * | * | * | * |
|  | Single factor | 733 (60.8) | 221 (62.1) | 90 (84.1) | 163 (48.9) | 98 (68.1) | 8 (80.0) | 64 (71.1) | 10 (100.0) | 20 (48.8) | * | * | 56 (88.9) |
| Medication change, % of all medication changes | Stop sulfonylureas | 40 (6.9) | 5 (4.0) | 9 (12.3) | 5 (3.1) | 8 (11.3) | 0 (0.0) | 6 (10.9) | 0 (0.0) | 0 (0.0) | * | * | * |
|  | Reduce sulfonylureas | 24 (4.1) | 6 (4.8) | 9 (12.3) | * | * | 0 (0.0) | * | 0 (0.0) | 0 (0.0) | * | 0 (0.0) | * |
|  | Stop insulin | 85 (14.7) | 15 (12.0) | * | 36 (22.4) | 17 (23.9) | 0 (0.0) | 5 (9.1) | * | * | * | * | * |
|  | Reduce insulin | 414 (71.4) | 96 (76.8) | 50 (68.5) | 113 (70.2) | 39 (54.9) | 5 (100.0) | 43 (78.2) | 9 (90.0) | 22 (91.7) | * | 8 (44.4) | 25 (73.5) |
|  | Changes to others | 111 (19.1) | 24 (19.2) | 14 (19.2) | 18 (11.2) | 16 (22.5) | * | 9 (16.4) | 0 (0.0) | 5 (20.8) | * | 10 (55.6) | 14 (41.2) |
| Recurrent episodes by admission | First episodes | 741 (61.5) | 227 (63.8) | 51 (47.7) | 142 (42.6) | 110 (76.4) | 10 (100.0) | 88 (97.8) | 6 (60.0) | 22 (53.7) | * | 34 (72.3) | 50 (79.4) |
|  | Recurrent episode occurring in same admission as first | 464 (38.5) | 129 (36.2) | 56 (52.3) | 191 (57.4) | 34 (23.6) | 0 (0.0) | * | * | 19 (46.3) | * | 13 (27.7) | 13 (20.6) |
| Hyperglycaemia post-treatment |  | 27 (2.2) | 5 (1.4) | * | 10 (3.0) | 7 (4.9) | 0 (0.0) | 0 (0.0) | 0 (0.0) | 0 (0.0) | * | * | * |
| Discharge outcome | Died | 273 (22.7) | 87 (24.4) | 19 (17.8) | 85 (25.5) | 51 (35.4) | * | 17 (18.9) | 0 (0.0) | 0 (0.0) | * | * | 9 (14.3) |
|  | Complex | 469 (38.9) | 162 (45.5) | 19 (17.8) | 120 (36.0) | 51 (35.4) | * | 31 (34.4) | * | 28 (68.3) | * | 20 (42.6) | 32 (50.8) |
|  | Simple | 373 (31.0) | 86 (24.2) | 56 (52.3) | 93 (27.9) | 31 (21.5) | 5 (50.0) | 41 (45.6) | 5 (50.0) | 11 (26.8) | * | 21 (44.7) | 20 (31.7) |
|  | Other | 90 (7.5) | 21 (5.9) | 13 (12.1) | 35 (10.5) | 11 (7.6) | 0 (0.0) | * | * | * | * | * | * |

** Excluding unknown not listed on hospital records.

Supplementary Table 2: Data on episodes – results between two similar centres A and D. All results given as n (%), where n represents the number of episodes.

|  | Centre | A | D | p |
| --- | --- | --- | --- | --- |
| Cohort |  | 356 | 144 |  |
| Sex | Female | 178 (50.0) | 86 (59.7) |  |
|  | Male | 178 (50.0) | 58 (40.3) |  |
| Ethnicity* | White | 228 (65.5) | 90 (68.2) |  |
|  | Asian | 77 (22.1) | 26 (19.7) |  |
|  | Black | 39 (11.2) | 15 (11.4) |  |
|  | Other/mixed | 4 (1.1) | 1 (0.8) |  |
| Age | Years, median (IQR) | 65 (48-76) | 68 (59-76) |  |
| CCI | Score, median (IQR) | 5 (4-8) | 5 (3-7) | 0.0035 |
| Diabetes | Type 1 | 113 (31.7) | 41 (28.5) |  |
|  | Type 2 | 237 (66.6) | 98 (68.1) |  |
|  | Other type | 6 (1.7) | 5 (3.5) |  |
| Antidiabetic medications | Insulin | 269 (75.6) | 97 (67.4) |  |
|  | Biguanides | 97 (27.2) | 43 (29.9) |  |
|  | Sulfonylureas | 25 (7.0) | 13 (9.0) |  |
|  | DPP4i | 44 (12.4) | 18 (12.5) |  |
|  | SGLT2i | 21 (5.9) | 5 (3.5) |  |
|  | GLP-1 | 2 (0.6) | 0 (0.0) |  |
|  | Thiazolidinediones | 0 (0.0) | 0 (0.0) |  |
|  | Antipsychotics | 17 (4.8) | 3 (2.1) |  |
| Event level | 2 | 260 (73.0) | 86 (59.7) | 0.004 |
|  | 3 | 96 (27.0) | 58 (40.3) |  |
| Cognitive impairment* | Yes | 150 (58.4) | 70 (66.0) |  |
|  | No | 107 (41.6) | 36 (34.0) |  |
| Precipitant | Fasting/missed meals | 201 (56.5) | 32 (22.2) | <0.0001 |
|  | Intercurrent illness | 112 (31.5) | 96 (66.7) | <0.0001 |
|  | Incorrect medications | 29 (8.1) | 11 (7.6) |  |
|  | Exercise | 0 (0.0) | 0 (0.0) |  |
|  | Unclear | 78 (21.9) | 28 (19.4) |  |
|  | Multiple | 57 (16.0) | 18 (12.5) |  |
|  | Single | 221 (62.1) | 98 (68.1) |  |
| Furthest line treatment reached | Not fully documented | 49 (13.8) | 26 (18.1) |  |
|  | Oral glucose | 97 (27.2) | 23 (16.0) | 0.0077 |
|  | IV Dextrose | 196 (55.1) | 85 (59.0) |  |
|  | Glucagon | 14 (3.9) | 10 (6.9) |  |
| Total number of episodes where treatment has been used* | Oral glucose | 209 (73.3) | 61 (55.0) | 0.0007 |
|  | IV Dextrose | 208 (58.6) | 95 (66.0) |  |
|  | Glucagon | 14 (3.0) | 10 (5.8) |  |
| Hyperglycaemia | Yes | 5 (1.4) | 7 (4.9) | 0.0455 |
|  | No | 351 (98.6) | 137 (95.1) |  |
| Episodes outcome | Care continued as inpatient | 332 (93.3) | 120 (83.3) | 0.0012 |
|  | Sent to ITU | 6 (1.7) | 4 (2.8) |  |
|  | Sent home | 0 (0.0) | 1 (0.7) |  |
|  | Death | 18 (5.1) | 19 (13.2) | 0.0039 |
| Length of stay from episode start | Days, median (IQR) | 9 (3-19) | 14 (4-34) | 0.0286 |
| Discharge outcome | Died | 87 (24.4) | 51 (35.4) | 0.0151 |
|  | Complex | 162 (45.5) | 51 (35.4) | 0.0457 |
|  | Simple | 86 (24.2) | 31 (21.5) |  |
|  | Other | 21 (5.9) | 11 (7.6) |  |
| Adjustment | Medications change | 125 (35.1) | 71 (49.3) | 0.0045 |
|  | Specialist team | 62 (17.4) | 44 (30.6) | 0.0016 |
|  | Palliative care | 86 (24.2) | 30 (20.8) |  |
|  | CGM started | 10 (2.8) | 1 (0.7) |  |
|  | Glucagon prescription | 1 (0.3) | 0 (0.0) |  |
| Medications change | Stop sulfonylureas | 5 (4.0) | 8 (11.3) |  |
|  | Reduce sulfonylureas | 6 (4.8) | 3 (4.2) |  |
|  | Stop insulin | 15 (12.0) | 17 (23.9) | 0.0432 |
|  | Reduce insulin | 96 (76.8) | 39 (54.9) | 0.0022 |
|  | Changes to others | 24 (19.2) | 16 (22.5) |  |
| Recurrent episodes by admission | First episodes | 227 (63.8) | 110 (76.4) | 0.0062 |
|  | Recurrent episode occurring in same admission as first | 129 (36.2) | 34 (23.6) |  |

* Excluding unknowns not listed on hospital records.

QR, interquartile range; CCI, Charlson Comorbidity Index; DDP4i; dipeptidyl peptidase-4 inhibitors; SGLT2i, Sodium-Glucose Cotransporter-2 inhibitor; GLP-1, Glucagon-like peptide-1 mimetic; IV, intravenous; ITU, intensive treatment unit; CGM, continuous glucose monitoring.

Supplementary Table 3: Baseline data on episodes - all results by IHSG level. All results given as n (%), where n represents the number of episodes.

|  | IHSG Level | Level 2 | Level 3 | p |
| --- | --- | --- | --- | --- |
| Cohort |  | 896 (74.4) | 309 (25.6) |  |
| Sex | Female | 509 (56.8) | 136 (44.0) | 0.0001 |
|  | Male | 387 (43.2) | 173 (56.0) |  |
|  | Ratio F:M | 1:1.315 | 1.272:1 |  |
| Ethnicity* | White | 566 (68.7) | 211 (74.3) |  |
|  | Asian | 153 (18.6) | 48 (16.9) |  |
|  | Black | 93 (11.3) | 23 (8.1) |  |
|  | Other/mixed | 12 (1.5) | 2 (0.7) |  |
| Diabetes | Type 1 | 287 (32.0) | 71 (23.0) | 0.0007 |
|  | Type 2 | 591 (66.0) | 238 (77.0) |  |
|  | Other | 18 (2.0) | 0 (0.0) |  |
| Age | Median (IQR) | 74 (58-83) | 70 (60-79) | 0.0167 |
| CCI | Median (IQR) | 6 (4-7) | 6(4-8) |  |
| Antidiabetic medications | Insulin | 715 (79.8) | 219 (70.9) | 0.0015 |
|  | Biguanides | 279 (31.1) | 82 (26.5) |  |
|  | Sulfonylureas | 79 (8.8) | 28 (9.1) |  |
|  | DPP4i | 100 (11.2) | 59 (19.1) | 0.0006 |
|  | SGLT2i | 67 (7.5) | 15 (4.9) |  |
|  | GLP-1 | 11 (1.2) | 3 (1.0) |  |
|  | Thiazolidinediones | 3 (0.3) | 1 (0.3) |  |
|  | Antipsychotics | 54 (6.0) | 27 (8.7) |  |
| Cognitive impairment* |  | 98 (21.4) | 309 (100.0) | <0.0001 |

*Excluding unknown not listed on hospital records.

IQR, interquartile range; CCI, Charlson Comorbidity Index; CGM, continuous glucose monitoring; DDP4i, dipeptidyl peptidase-4 inhibitors; SGLT2i, sodium-glucose cotransporter-2 inhibitor; GLP-1, glucagon-like peptide-1mimetic.

Supplementary Table 4: Data on episodes - all results by episode type (first episode or recurrent). All results given as n (%), where n represents the number of episodes.

|  | Episode type | First episode | Recurrent episode | p |
| --- | --- | --- | --- | --- |
| Cohort |  | 741 (61.5) | 464 (38.5) |  |
| Sex | Female | 341 (46.0) | 304 (65.5) | <0.0001 |
|  | Male | 400 (54.0) | 160 (34.5) |  |
| Ethnicity* | White | 482 (71.0) | 295 (68.8) |  |
|  | Asian | 125 (18.4) | 76 (17.7) |  |
|  | Black | 61 (9.0) | 55 (12.8) | 0.0444 |
|  | Other/mixed | 11 (1.6) | 3 (0.7) |  |
| Diabetes | Type 1 | 193 (26.0) | 165 (35.6) | 0.0006 |
|  | Type 2 | 536 (72.3) | 293 (63.1) |  |
|  | Other | 12 (1.6) | 6 (1.3) |  |
| Age | Years, median (IQR) | 72 (59-82) | 75 (63-85) | 0.0024 |
| CCI | Score, median (IQR) | 6 (4-7) | 6 (5-8) | 0.0002 |
| Antidiabetic medications | Insulin | 547 (73.8) | 387 (83.4) | <0.0001 |
|  | Biguanides | 251 (33.9) | 110 (23.7) | 0.0002 |
|  | Sulfonylureas | 72 (9.7) | 35 (7.5) |  |
|  | DPP4i | 99 (13.4) | 60 (12.9) |  |
|  | SGLT2i | 58 (7.8) | 24 (5.2) |  |
|  | GLP-1 | 10 (1.3) | 4 (0.9) |  |
|  | Thiazolidinediones | 3 (0.4) | 1 (0.2) |  |
|  | Antipsychotics | 37 (5.0) | 44 (9.5) | 0.003 |
| Event level | 2 | 527 (71.1) | 369 (79.5) | 0.0011 |
|  | 3 | 214 (28.9) | 95 (20.5) |  |
| Cognitive impairment* | Yes | 283 (56.0) | 124 (47.5) | 0.0268 |
|  | No | 222 (44.0) | 137 (52.5) |  |
| Precipitant | Fasting/missed meals | 267 (36.0) | 223 (48.1) | <0.0001 |
|  | Intercurrent illness | 314 (42.4) | 225 (48.5) | 0.0429 |
|  | Incorrect medications | 110 (14.8) | 91 (19.6) | 0.0323 |
|  | Exercise | 0 (0.0) | 1 (0.2) |  |
|  | Unclear | 200 (27.0) | 98 (21.1) | 0.0235 |
|  | Multiple | 85 (11.5) | 90 (19.4) | 0.0011 |
|  | Single | 456 (61.5) | 276 (59.5) |  |
| Furthest line treatment used | None documented | 185 (25.0) | 121 (26.1) |  |
|  | Oral glucose | 215 (29.0) | 174 (37.5) | 0.0024 |
|  | IV Dextrose | 306 (41.3) | 155 (33.4) | 0.0062 |
|  | Glucagon | 35 (4.7) | 14 (3.0) |  |
| Total number of episodes treatment used* | Oral glucose | 369 (67.1) | 262 (76.6) | 0.0025 |
|  | Dextrose | 329 (44.4) | 165 (35.6) | 0.0026 |
|  | Glucagon | 35 (5.4) | 14 (3.5) |  |
| Hyperglycaemia | Yes | 20 (2.7) | 7 (1.5) |  |
|  | No | 721 (97.3) | 457 (98.5) |  |
| Episode Outcome | Care continued as inpatient | 669 (90.3) | 446 (96.1) | 0.0001 |
|  | Sent to ITU | 9 (1.2) | 3 (0.6) |  |
|  | Sent home | 4 (0.5) | 1 (0.2) |  |
|  | Death | 59 (8.0) | 14 (3.0) | 0.0004 |
| Length of stay from episode start | Days, median (IQR) | 7 (3-18) | 10 (5-24) | <0.0001 |
| Discharge outcome | Died | 160 (21.6) | 113 (24.4) |  |
|  | Complex | 260 (35.1) | 209 (45.0) | 0.0007 |
|  | Simple | 272 (36.7) | 101 (21.8) | <0.0001 |
|  | Other | 49 (6.6) | 41 (8.8) |  |
| Adjustment | Medications change | 351 (47.4) | 229 (49.4) |  |
|  | Specialist team | 212 (28.6) | 128 (27.6) |  |
|  | Palliative care | 117 (15.8) | 93 (20.0) |  |
|  | CGM started | 21 (2.8) | 5 (1.1) |  |
|  | Glucagon | 3 (0.4) | 0 (0.0) |  |
| Medications change, % of all medications changes | Stop sulfonylureas | 27 (7.7) | 0 (0.0) | <0.0001 |
|  | Reduce sulfonylureas | 16 (4.6) | 8 (3.5) |  |
|  | Stop insulin | 50 (14.2) | 35 (15.3) |  |
|  | Reduce insulin | 243 (69.2) | 171 (74.7) |  |
|  | Changes to others | 73 (20.8) | 38 (16.6) |  |

* Excluding unknowns not listed on hospital records.

IQR, interquartile range; CCI, Charlson Comorbidity Index; DDP4i, dipeptidyl peptidase-4 inhibitors; SGLT2i, Sodium-Glucose Cotransporter-2 inhibitor; GLP-1, Glucagon-like peptide-1 mimetic; IV, intravenous; ITU, intensive treatment unit; CGM, continuous glucose monitoring.

Supplementary Table 5: Data on admission - all results by admission type (first admission or recurrent admissions). All results given as n (%), where n represents the number of admissions.

| Admission type |  | Overall | First admission | Readmission | P (between first admission and readmissions) |
| --- | --- | --- | --- | --- | --- |
| Cohort |  | 739 (100.0) | 656 (88.8) | 83 (11.2) |  |
| Discharge outcome | Died | 159 (21.5) | 152 (23.2) | 7 (8.4) | 0.0016 |
|  | Complex | 257 (34.8) | 215 (32.8) | 42 (50.6) | 0.0021 |
|  | Simple | 276 (37.3) | 246 (37.5) | 30 (36.1) |  |
|  | Other | 47 (6.4) | 43 (6.6) | 4 (4.8) |  |
| Adjustment | Medication change | 348 (47.1) | 309 (47.1) | 39 (47.0) |  |
|  | Specialist team | 213 (28.8) | 195 (29.7) | 18 (21.7) |  |
|  | Palliative care | 113 (15.3) | 106 (16.2) | 7 (8.4) |  |
|  | CGM started | 21 (2.8) | 16 (2.4) | 5 (6.0) |  |
|  | Glucagon | 3 (0.4) | 3 (0.5) | 0 (0.0) |  |
| Medications change, % of medications changes | Stop sulfonylureas | 28 (8.0) | 25 (8.1) | 3 (7.7) |  |
|  | Reduce sulfonylureas | 12 (3.4) | 12 (3.9) | 0 (0.0) |  |
|  | Stop insulin | 51 (14.7) | 45 (14.6) | 6 (15.4) |  |
|  | Reduce insulin | 240 (69.0) | 211 (68.3) | 29 (74.4) |  |
|  | Changes to others | 3 (0.9) | 3 (1.0) | 0 (0.0) |  |

CGM, continuous glucose monitoring.
